# Dataset of metagenomic profiles of human gut microbiome from frozen fecal samples sequenced using Illumina and ONT chemistries

**DOI:** 10.1016/j.dib.2024.110961

**Published:** 2024-09-19

**Authors:** Gauraw Kumar, Punyasloke Bhadury

**Affiliations:** Integrative Taxonomy and Microbial Ecology Research Group, Department of Biological Sciences, Indian Institute of Science Education and Research Kolkata, Mohanpur, Nadia 741246 West Bengal, India

**Keywords:** Human fecal sample, Next-generating sequencing, Illumina, Oxford Nanopore Technologies

## Abstract

The data presented in this study are metagenomic profiles of human gut microbiome deduced from frozen fecal samples using two different sequencing chemistries namely, Illumina and Oxford Nanopore Technologies (ONT). The generated data is obtained from genomic DNA extracted from frozen fecal samples collected from a healthy individual on Day 3, Day 5, Day 9, Day 12, and Day 30, in addition to Day 1 (unfrozen). The metagenomic sequence data have been deposited at NCBI SRA as BioProject PRJNA827663. The taxonomic annotation undertaken using MG-RAST showed relative abundance of bacteria represented by different taxonomic levels varied significantly based on two sequencing chemistries. There was distinct temporal variation in the relative abundance of bacteria at different taxonomic levels based on the day of extraction of genomic DNA. This dataset can be used to study differences in functional profiles of human gut microbiome using different sequencing technologies. Moreover, generated data can aid in selection of most appropriate sequencing chemistry to study future human gut microbiome studies based on the appropriate preservation method of fecal samples.

Specifications Table

**Table:** 

Subject	Biology
Specific subject area	Metagenomics
Data format	Raw and analyzed
Type of data	DNA sequences and Figure
Data collection	Metagenomic sequencing of human fecal samples using Illumina and Oxford Nanopore Technologies (ONT) chemistries
Data source location	Integrative Taxonomy and Microbial Ecology Research Group, Department of Biological Sciences, Indian Institute of Science Education and Research Kolkata, Mohanpur, Nadia, 741246, West Bengal, India.
Data accessibility	Repository name: NCBI SRA Data identification number: BioProject PRJNA827663, BioSamples SAMN38203146, SAMN38203147, SAMN27616101, SAMN27616100, SAMN27616099, SAMN27616098, SAMN27616097 and SAMN27616096 Direct URL to data: https://www.ncbi.nlm.nih.gov/bioproject/PRJNA827663

## 1 Value of the Data

• The dataset provides comparison of two types of sequencing chemistries applied on genomic DNA extracted from human fecal samples (unfrozen and frozen over 30 days) from a healthy individual.
• The data provides valuable information on the selection of most appropriate sequencing chemistry to map temporal patterns of human gut microbiome taxonomic profiles from fecal samples.
• The generated raw sequence data can be used for downstream computational biology approaches towards elucidation of functional profiling mediated by gut microbiome and linking to One Health.

## 2 Background

Next-generation sequencing (NGS) has revolutionized human gut microbiome studies globally and have significantly improved our understanding of how microbiomes impact health and disease. There has been an increasing global interest to map community composition of human gut microbiome led by scientists and clinicians for effective management of public health [1] as well as to achieve One Health perspective. However, differences in methodology adopted across a large number of studies make it difficult to compare findings among and between the studies for broader understanding of human health across geographical scales. While some studies suggest caution in choosing appropriate sequencing chemistry owing to differences in generation of data and quality; others have reported comparable reproducibility and treatment effects on microbiome community trends such as diversity [[2], [3], [4]]. In this study same broad methodology and bioinformatics pipeline were applied for human fecal samples to examine if two different sequencing chemistries give different compositional information of human gut microbiome. This study also explored how human gut microbiome changes temporally based on the use of preservation method such as freezing of fecal samples.

## 3 Data Description

**Fig. 1:**
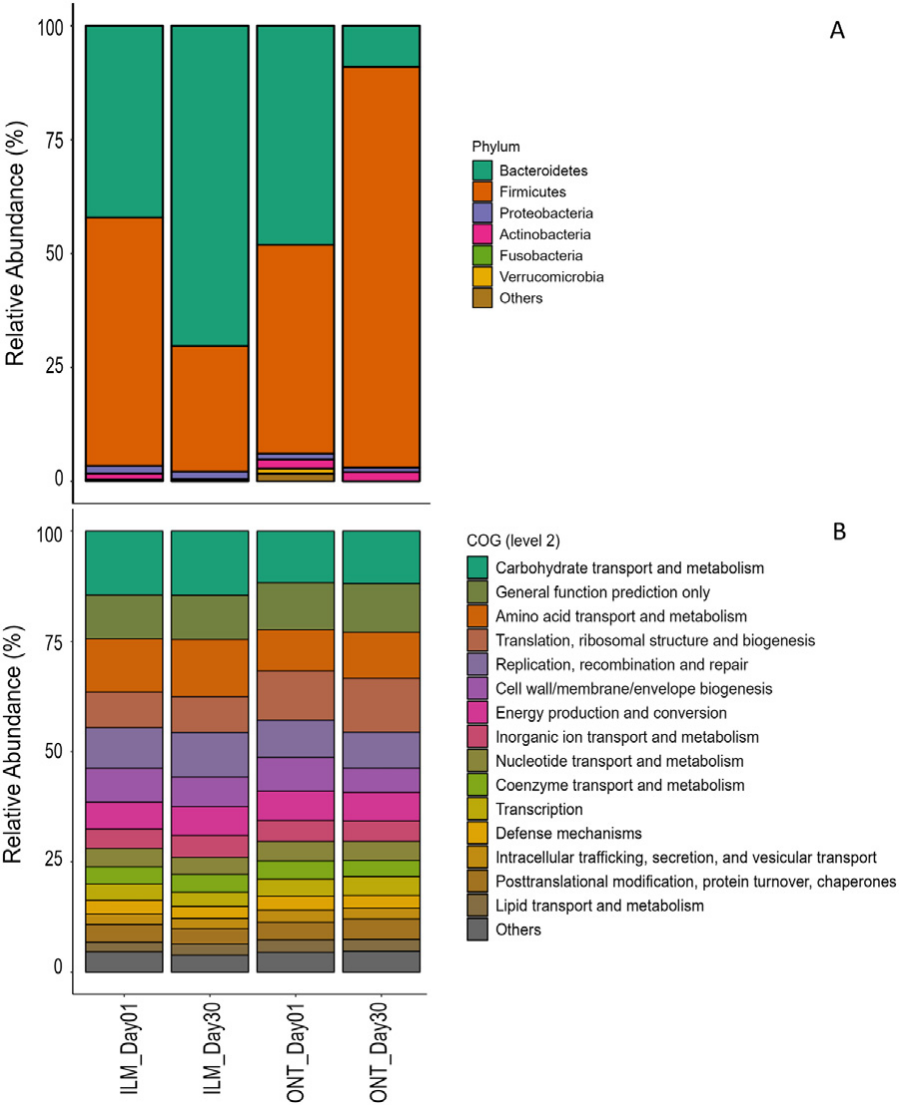
Relative abundance of bacterial phyla and functional profiles (COG) generated from datasets using Illumina and ONT sequencing chemistries.

**Fig. 2:**
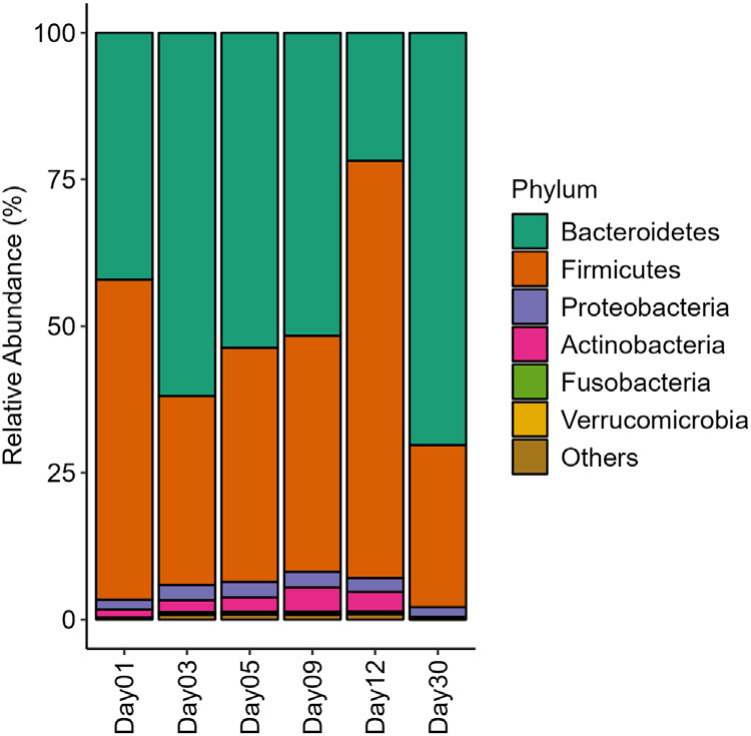
Temporal variations in structure of human gut microbiome (bacterial) communities at the phylum level for Day 1, Day 3, Day 5, Day 9, Day 12, and Day 30 following preservation based on Illumina sequencing chemistry.

The datasets comprised of raw data generated through metagenomic sequencing of human fecal samples collected from a healthy individual, aged 25 years, from India. The genomic DNA was extracted from collected fecal samples. The extraction of genomic DNA was undertaken on Day 1, Day 3, Day 5, Day 9, Day 12, and Day 30 of the collection of fecal sample. The genomic DNA extracted on Day 1 and Day 30 were sequenced on both MiniSeq and MinION™ platforms based on Illumina and Oxford Nanopore Technologies (ONT) sequencing chemistries respectively. Additionally, genomic DNA for Day 3, Day 5, Day 9, and Day 12 were sequenced only using Illumina sequencing chemistry. All the data files (reads in FASTQ format) have been deposited in NCBI SRA database under the project accession number PRJNA827663. The MiniSeq platform generated a total of 5,43,939 reads and the MinION™ platform generated a total of 2,10,714 reads. The short reads generated on the MiniSeq platform were of high quality and did not require trimming. The long reads generated on the MinION™ platform were processed to remove the adapter. Upon trimming, reads that were less than 100 bp were removed and only 2,07,500 reads were considered for downstream processing. A total of 76.9% reads had Q-score > 10 and median read length of 595 and 1263 bp for Day 1 and Day 3 genomic DNA respectively sequenced using ONT approach. Using the MG-RAST pipeline (https://www.mg-rast.org/), sequences were taxonomically assigned to 9 phyla, 15 classes, and 67 genera representing the microbiome communities. The most abundant bacterial phyla identified were Bacteroidetes and Firmicutes. Functional analyses using COG, NOG, and KO databases at all levels were also performed using MG-RAST pipeline. The relative abundance of bacterial taxa at the phylum level and level 2 distribution for COG database based on Illumina and ONT chemistries have been shown in Fig.1. The temporal variations in human gut microbiome composition at the phylum level for Day 1, Day 3, Day 5, Day 9, Day 12, and Day 30 based on preservation periods sequenced using Illumina sequencing chemistry have been shown in Fig.2.

## 4 Experimental Design, Materials and Methods

### 4.1 Sample collection and DNA extraction

The stool sample was collected with informed consent from a healthy individual aged 25 years in a sterile container (Tarsons, India). The sample was stored at −20 °C immediately within 30 min of collection and the same was kept frozen throughout the duration of experiment. Only one sample was not frozen and immediately subjected to genomic DNA extraction. The genomic DNA extraction was performed using QIAamp Fast Stool DNA Minikit as per the manufacturer's instructions (Qiagen). The genomic DNA was extracted on Day 1, Day 3, Day 5, Day 9, Day 12, and Day 30 of the collection from frozen stool sample. The yield and quality were analysed by 1 % agarose gel electrophoresis and Nanodrop 2000 Spectrophotometer (Thermo Fisher Scientific, USA). The dsDNA concentration was also determined using Qubit 3.0 Fluorometer (Thermo Fisher Scientific, USA).

### 4.2 Library preparation and sequencing

The library for Illumina sequencing chemistry was prepared using Illumina DNA Prep kit and Nextera DNA CD Indexes (Illumina, USA) following manufacturer's instructions. The prepared library was checked using a 2100 Bioanalyzer (Agilent Technologies, USA). Metagenomic paired-end sequencing was performed using the MiniSeq high output 150 cycle kit (Illumina, USA) on MiniSeq platform (Illumina, USA). The library preparation based on Oxford Nanopore Technologies (ONT), was performed using the Ligation Sequencing Kit (SQK-LSK109; Oxford Nanopore Technologies, Oxford, UK) and PCR Barcoding Kit (EXP-PCR096; Oxford Nanopore Technologies, Oxford, UK). The sequencing was performed in MinION™ platform using SpotON Flowcell R9.4 (FLO-MIN 106).

### 4.3 Data analyses

The quality of the paired-end reads generated using Illumina sequencing chemistry were checked using FastQC. The paired-end reads were merged using FLASH (Galaxy Version 1.2.11.4) with a minimum overlap of 10 bp and a maximum overlap of 65 bp between the two reads using 0.25 as the maximum mismatch density [5]. The reads generated using ONT were merged using “Concatenate multiple datasets” tool on the Galaxy platform (usegalaxy.eu). Adapter trimming for the ONT reads were undertaken using Porechop [6] and quality control was undertaken using Nanoplot [7] and Filtlong [8]. The merged reads were analysed on MG-RAST (version 4.0) pipeline after removing artificial replication reads and host-specific *H. sapiens* NCBI v36 species sequence. Taxonomic classification of reads was given using the “Best Hit” classification method and taxonomy was assigned using SILVA database. Only bacterial taxa were considered in this study. The statistical tests were performed on R 3.6.2 (R Core Team 2021) using ggplot2 [9], reshape2 [10], gplots [11] and vegan [12].

## Limitations

The major limitation of this study is the limited sample size.

## Ethics Statement

Ethical clearance for this study was obtained from the Institute Ethics Committee of Indian Institute of Science Education and Research Kolkata (IISER) (IISER/IEC/2022/03). Informed consent was obtained and carried out in accordance with the Declaration of Helsinki.

## Credit Author Statement

**Gauraw Kumar**: Conceptualization, Methodology, Software, Writing, Data curation, Original draft preparation. **Punyasloke Bhadury**: Conceptualization, Supervision, Writing, Validation.

## Data Availability

How does gut microbial community structure and its functional profile changes when human fecal samples are fixed in different fixative in 30 days period (Original data) (NCBI).
